# High Maternal Total Cholesterol Is Associated With No-Catch-up Growth in Full-Term SGA Infants: The Japan Environment and Children’s Study

**DOI:** 10.3389/fendo.2022.939366

**Published:** 2022-07-14

**Authors:** Kayo Kaneko, Yuki Ito, Takeshi Ebara, Sayaka Kato, Taro Matsuki, Hazuki Tamada, Hirotaka Sato, Shinji Saitoh, Mayumi Sugiura-Ogasawara, Hiroshi Yatsuya, Michihiro Kamijima

**Affiliations:** ^1^ Department of Occupational and Environmental Health, Graduate School of Medical Sciences, Nagoya City University, Nagoya, Japan; ^2^ Department of Pediatrics and Neonatology, Graduate School of Medical Sciences, Nagoya City University, Nagoya, Japan; ^3^ Department of Obstetrics and Gynecology, Graduate School of Medical Sciences, Nagoya City University, Nagoya, Japan; ^4^ Department of Public Health and Health Systems, Graduate School of Medicine, Nagoya University, Nagoya, Japan

**Keywords:** catch-up growth, developmental origins of health and disease (DOHaD), prospective cohort, small for gestational age (SGA), total cholesterol (TC)

## Abstract

**Objectives:**

Infants born small for gestational age (SGA) with no catch-up growth (No-CU) are at high risk of intellectual and developmental disabilities. However, factors leading to No-CU among SGA infants are unclear. This study aimed to examine the association between maternal total cholesterol (TC) in mid-pregnancy and No-CU at 3 years among full-term SGA infants.

**Study Design:**

The Japan Environment and Children’s Study (JECS) is a nationwide prospective birth cohort study. We extracted a total of 2,222 mothers and full-term SGA infants (length and/or weight <‐2 standard deviation [SD]) without congenital abnormalities from the original JECS cohort comprising a total of 104,062 fetal records. According to the distribution of maternal TC in the entire cohort, participants were classified into nine groups per each fifth percentile with the 20th–79th percentiles (204–260 mg/dl) as the reference group. No-CU was defined by a Z-score of height at 3 years <‐2 SD according to the growth standard charts for Japanese children. Multivariable-adjusted logistic regression models were carried out using multiple imputations. Additionally, a multiple-adjusted restricted cubic spline model was performed in the complete dataset.

**Results:**

A total of 362 (16.3%) children were No-CU at 3 years. After adjusting for the Z-score of birth weight, age of mother, smoking status, weight gain during pregnancy, breastfeeding and meal frequency at 2 years, and parents’ heights, the odds ratio (95% confidence intervals) of No-CU was 2.95 (1.28–6.80) for children whose maternal TC levels were in the highest category (≥294 mg/dl), compared to the reference group. A multiple-adjusted restricted cubic spline model showed a non-linear trend of the significant association between high maternal TC and No-CU (p for linear trend = 0.05, p for quadratic trend <0.05).

**Conclusion:**

High maternal TC at mid-pregnancy was associated with No-CU among SGA infants. Such infants should be carefully followed up to introduce appropriate growth hormonal treatment. The findings may support previous animal experimental studies which indicated that maternal high-fat diet exposure induces impairment of growth and skeletal muscle development in the offspring. Future studies are required to elucidate the detailed mechanism.

## Introduction

Infants born small for gestational age (SGA) are at a high risk of long-lasting intellectual and psychological impairments such as low cognitive function, aggressive behaviors, and attention-deficit hyperactivity disorder (1–6). Approximately 85–90% of SGA infants catch up in height above -2 standard deviations (SD) during the first 2 years of life (5, 7–11). SGA infants with no catch-up growth (No-CU) have a particularly high risk of future intellectual and developmental disabilities, and clinical intervention is required to maintain growth retardation (12, 13). Since growth hormone (GH) and insulin-like growth factor (IGF) I play important roles for early brain growth and maturation in infancy (14), several studies reported that GH treatment induced catch-up growth in infants born SGA and concurrently improved intellectual performances (15–17), thereby underscoring the need for earlier diagnosis and introduction of GH treatment (8, 14, 18). Therefore, accurate surveillance of SGA infants to detect early signs of No-CU is needed to guide the appropriate introduction of GH treatment (8, 19, 20). In addition, identification of prenatal factors that would lead to or be a marker of No-CU may contribute to preventing growth retardation among SGA infants.

Since the maternal supply of cholesterol to the fetus plays an important role in the development of the fetal nervous and vascular system (21–23), the maternal blood level of total cholesterol (TC) is linearly associated with the infant’s birth size (24–27). In contrast, findings from recent animal experimental studies indicated that maternal high-fat diet induces impairment of lung development and function (28, 29), skeletal muscle (30), and/or bone mass development (31–33) and growth pattern (34) in the offspring. Although maternal lipids in the critical fetal period can influence infant growth, no study has investigated whether the maternal lipid blood level is associated with No-CU among SGA infants.

Previously, we had shown that maternal TC levels in mid-pregnancy were associated with SGA or large for gestational age in the Japan Environment and Children’s Study (JECS), a Japanese nationwide birth cohort study (35). As a subsequent investigation, this study aimed to examine the association between maternal TC in mid-pregnancy (14th–27th gestational weeks) and No-CU at 3 years among infants born SGA at term in JECS.

## Materials and Methods

The JECS was established in 2011 with 15 Regional Centers located across Japan (36–38). This is an ongoing nationwide birth cohort study to examine the effect of environmental factors and medical, psychosocial, and lifestyle conditions during pregnancy and childhood on the infant’s health and development at birth and later in life. The details of the study design and profile have been described previously (36, 38). Briefly, between January 2011 and March 2014, the cooperating health care providers and/or research coordinators" into Co-operating health care providers and/or Research Coordinators recruited pregnant women during the first trimester and/or local government office-issued home-based records for mother and child, namely, Maternal and Child Health Handbooks (the Handbook is an official booklet given to all expecting mothers in Japan to receive municipal services for pregnancy, delivery, and childcare, and the related medical records are kept at home). The eligibility criteria for participants were (i) living in the study area at the time of recruitment (i.e., at any of the 15 regional centers throughout Japan) and expected to continue to reside in Japan for the foreseeable future; (ii) an expected delivery between August 1, 2011, and mid-2014; and (iii) understanding the Japanese language in order to complete the self-administered questionnaire.

The study protocol of the JECS was reviewed and approved by the Ministry of the Environment’s Institutional Review Board on Epidemiological Studies and the ethics committees of all participating institutions. All participants in the present study provided written informed consent. Data for the current analyses were extracted from the dataset released in October 2019 (dataset -jecs-ta-20190930-qsn).

### Study Participants

**Figure 1:**
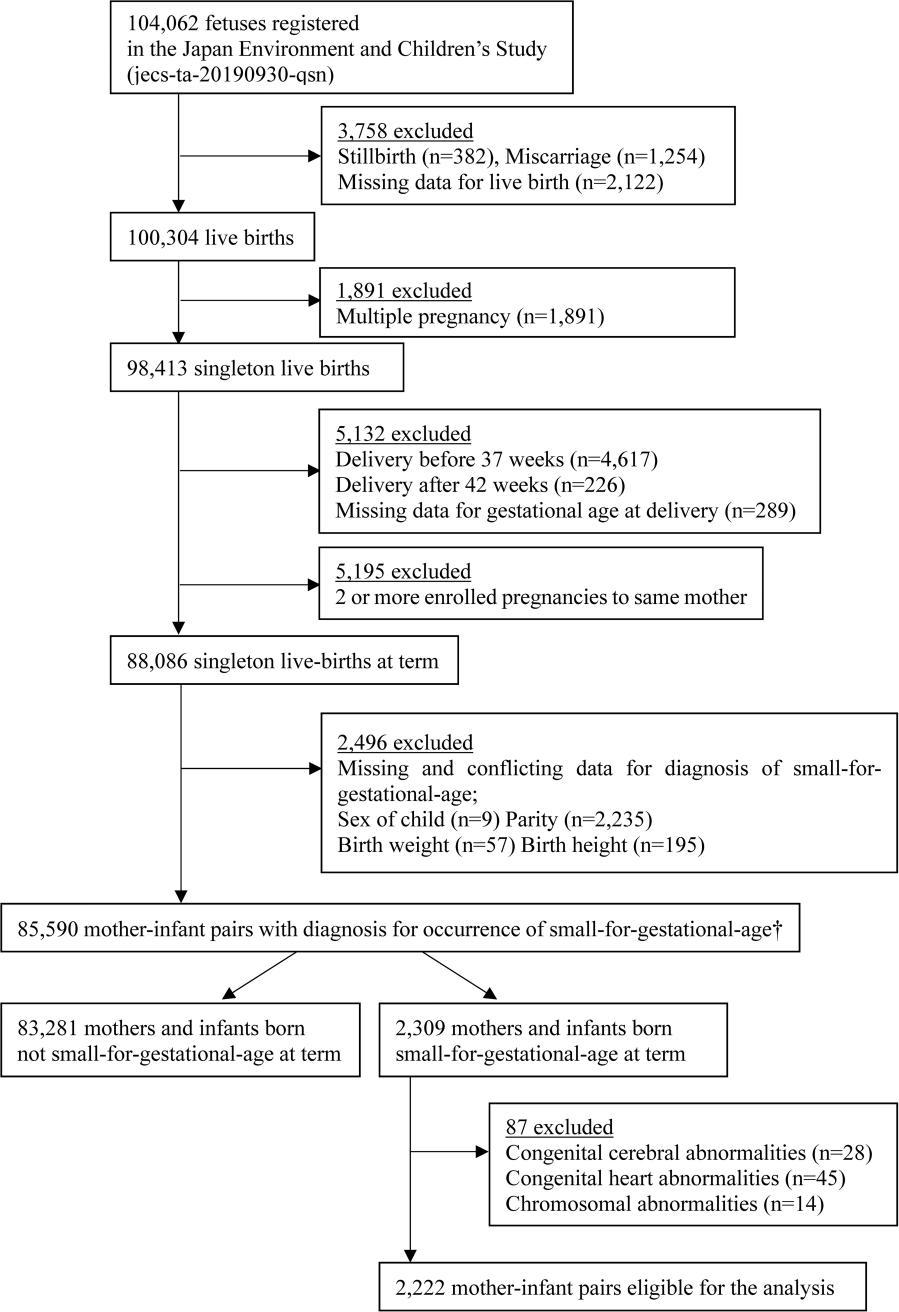
Study enrollment flowchat.

The present study involved a total of 2,222 mothers with infants born SGA (length and/or weight <-2 SD) who were extracted from the original JECS cohort comprising a total of 104,062 fetal records. First, this study was restricted to 88,086 mother-and-infant pairs with singleton live births at term. Second, 2,235 mother-and-infant pairs were excluded because of missing or conflicting data for SGA diagnosis. As the present study focused on SGA infants, we excluded 83,281 mothers and non-SGA infants. Finally, 87 cases with congenital cerebral, heart, and/or chromosomal abnormalities were excluded, leaving 2,222 mother-and-SGA-infant pairs eligible for the present analysis (**Figure 1**).

### Bioassay Measurements

Non-fasting blood samples were collected from pregnant women at early and mid-pregnancy. A contract clinical laboratory (SRL, Inc., a commercial laboratory in Tokyo, Japan) assayed and analyzed enzymatically serum blood TC levels at mid-pregnancy using a 7700 clinical chemistry/immunoassay hybrid analyzer (Hitachi High-Technologies Co., Ltd., Tokyo, Japan) (39).

### Explanatory Variables

Raw maternal TC values and gestational age (days) at the time of blood sampling were significantly positively correlated. Therefore, the analysis was adjusted for gestational age at the time of blood sampling by residual methods (35, 40). The optimal cutoff values for the maternal TC are still unclear, although a significantly higher risk of adverse birth-related outcomes was observed in the maternal TC <3rd (25) and ≥95th (27) percentiles according to previous studies. We categorized the adjusted maternal TC into nine groups according to the distribution of the entire cohort per the following percentile ranges, <5th, 5–9th, 10th–14th, 15th–19th, 20th–79th (reference group), 80th–84th, 85th–89th, 90th–94th, and ≥95th, to explore the reference value of the maternal TC at mid-pregnancy for No-CU among SGA infants.

### Definition of SGA and Ascertainment of No-CU

The Co-operating health care providers and/or Research Coordinators in 15 Regional Centers transcribed gestational age at birth and data on neonatal anthropometric measurements from the medical records. To determine the gestational age in weeks and days, ultrasonography during early pregnancy and/or estimation from the last menstrual period were used. The Z-score of birth weight or length for gestational age was calculated based on Japanese neonatal anthropometric charts (41).

We adopted two SGA definitions. First, SGA was defined as length and/or weight <-2 SD at birth according to the criterion of the global consensus statement for growth hormone (GH) treatment (8). As a secondary analysis, we defined SGA as height and/or weight at birth <-2 SD plus both height and weight at birth less than the 10th percentile based on the clinical definition of GH treatment in Japan (19).

Children’s height at 3 years was reported by their mothers in a self-administrated questionnaire which was distributed around the third birthday (3 years of age) of the children. In Japan, all municipal and regional administrative offices are legally required to provide a free medical health checkup for 1.5- and 3-year-old children, and approximately 95% of the eligible children are examined. In addition, the “Manual for evaluation of infant and child physical development” indicates that height of children aged 2 years or more must be measured by medical professionals in the standing position to the nearest 0.1 cm (42). Thus, almost all mothers could transcribe the results of the free medical health checkup for their children at 1.5 and 3 years of age from the Maternal and Child Health Handbooks to the questionnaire.

No-CU was defined by a Z-score of height at 3 years <- 2 SD according to the growth standard charts for Japanese children (9, 43), since a previous study reported that the catch-up rate did not increase after 3 years of age in Japanese children (10).

### Covariates

Socioeconomic data and lifestyle-related information were self-reported during the early and mid-period of pregnancies (44). Anthropometric measurements, medical history, and medical information during pregnancy and at delivery were transcribed from the medical records by physicians, midwives/nurses, and/or Research Coordinators. The Z-score of birth weight was calculated based on Japanese neonatal anthropometric charts (41) using sex of child, parity, gestational age at birth, and birth weight. The father’s height was self-reported or reported by the partner. Breastfeeding status and frequency of meal or snack intake at 2 years of age were reported by the mother.

The Z-score of birth weight, the mother’s age at delivery, and weight gain during pregnancy were used as continuous variables. Smoking status (45) was classified as “Never,” “Previously did, but quit before recognizing current pregnancy,” “Previously did, but quit after finding out current pregnancy,” and “Yes, I still smoke.” Breastfeeding (46) status at 2 years was categorized as “currently continued breastfeeding,” “stopped breastfeeding before,” and “never breastfed.” Frequency of meal or snacks per day at 2 years was dichotomized as “less than 3 times” and “3 times or more.” Study areas were divided into the location of the 15 regional centers, which included “Hokkaido,” “Miyagi,” “Fukushima,” “Chiba,” “Kanagawa,” “Koshin,” “Toyama,” “Aichi,” “Kyoto,” “Osaka,” “Hyogo,” “Tottori,” “Kochi,” “Fukuoka,” and “South Kyushu/Okinawa”.

These covariates were selected based on current biological and epidemiological knowledge related to postnatal growth or a strategy of selecting variables according to a certain level of statistical significance (e.g., p < 0.1 or p < 0.05) in the univariable analyses. The accuracy of modeling was assessed using discrimination and goodness of fit. A fully adjusted model discriminated No-CU reasonably well, with a rate of correct classifications of 84.2% and p-value of 0.52 for goodness of fit by Hosmer–Lemeshow test.

### Statistical Analysi*s*

In total, 1,088 records of 2,222 subjects (49.0%) were incomplete. Missing values for mother’s height (0.1%), smoking status (2.7%), weight gain during pregnancy (2.4%), breastfeeding status (16.7%), meal frequency (21.4%) at 2 years, father’s height (15.1%), No-CU occurrence at 3 years (27.4%), and maternal TC categories at mid-pregnancy (16.5%) were imputed using the Missing Values option in SPSS. The results using the 20 imputed data sets were combined as average, and standard errors were adjusted to reflect both within-imputation variability and between-imputation variability (47). Because the multiple-imputation procedure was performed under the assumption that missing data were missing at random, we performed complete data analysis restricted to those with all measurements (n = 1,134) as a sensitivity analysis.

The characteristics of participants according to the occurrence of No-CU are reported as means and SD for continuous variables. Differences in continuous data were tested using an analysis of variance. Categorical variables are described as frequency (%) and analyzed by χ^2^ tests.

Multivariable adjusted odds ratios (ORs) and the 95% confidence intervals (CIs) of the gestational age-adjusted maternal TC categories for No-CU at 3 years were estimated using a logistic regression model. The first model was adjusted for Z-score of birth weight and maternal age at delivery (years) (model 1). The second model included variables adjusted for model 1 plus maternal weight gain during pregnancy (kg), smoking status during pregnancy (Never; Previously did, but quit before recognizing current pregnancy; Previously did, but quit after finding out current pregnancy; and Yes, I still smoke), and study areas (Hokkaido, Miyagi, Fukushima, Chiba, Kanagawa, Koshin, Toyama, Aichi, Kyoto, Osaka, Hyogo, Tottori, Kochi, Fukuoka, and South Kyushu/Okinawa) (model 2). The third model additionally adjusted for breastfeeding status at 2 years (currently continued breast feeding, stopped breast feeding before, never breastfed) and the frequency of meals or snacks per day at 2 years (<3 times, ≥3 times) (model 3). The final model was further adjusted for maternal and paternal height (cm) (model 4).

In an attempt to examine the dose–response relationship, we modeled each maternal TC percentile category as the following value provided in the parentheses and treated it as a continuous variable; <5th (-4), 5th–9th (-3), 10th–14th (-2), 15th–19th (-1), 20th–79th (0), 80th–84th (1), 85th–89th (2), 90th–94th (3), and ≥95th (4).

Additionally, we constructed a restricted cubic spline model. In the model, maternal TC in mid-pregnancy was included as a restricted cubic spline using six knots at prespecified locations according to the percentiles of the distribution, the 5th, 10th, 25th, 75th, 90th, and 95th percentiles, and was adjusted for all covariates in model 4. The curves show ORs compared to the median value of the maternal TC (=227 mg/dl) in the present analysis dataset. In addition, tests for linear and quadratic trends were performed.

All statistical analyses were performed using IBM SPSS Statistics for Windows software (version 27.0; IBM, Armonk, NY, USA) and R (version 3.4.3; Vienna, Austria) for Windows (http://cran.r-project.org/). Statistical significance was set at p < 0.05.

## Results

### Characteristics of Participants

The infants in the present study had a mean (SD) gestational age of 39.2 (1.1) weeks, length at birth of 45.3 (0.9) cm, and birth weight of 2436.9 (290.9) g; 42.2% of infants were males. The women in the present study had a mean (SD) age of 31.0 (5.1) years; 50.8% were nulliparous. The mean (SD) gestational age at the blood sampling of maternal TC was 22.8 (4.1) weeks.

Compared to children with catch-up growth at 3 years, Z-score of birth weight, the maternal weight gain during pregnancy, parents’ heights, and the rate of children who had a meal or snack ≥3 times per day at 2 years were significantly lower in No-CU children. In addition, mothers of children with catch-up growth at 3 years tended to be nulliparous and practice breastfeeding, although these results were not significant. Smoking status during pregnancy did not significantly differ according to the occurrence of No-CU (**Table 1**).

**Table 1:** Characteristics of participants by occurrence of catch-up growth at 3 years among infants born small for gestational age^†^ at term (n = 2,222).

	Total	Catch-up growth at 3 years	No catch-up growth at 3 years	*P* value
	(n = 2,222)	(n = 1,860)	(n = 362)	
Sex of child (male)	937 (42.2)	783 (42.1)	155 (42.8)	0.84
Gestational age at birth (weeks)	39.2 (1.1)	39.2 (1.2)	39.0 (1.2)	<0.01
Z-score of birth length	-2.0 (1.0)	-1.9 (1.0)	-2.0 (0.9)	0.08
Z-score of birth weight	-1.8 (0.8)	-1.8 (0.9)	-2.0 (0.9)	<0.05
Age of mother at delivery (years)	31.0 ± 5.1	31.0 ± 5.3	30.7 ± 5.7	0.18
Parity (≥1)	1,093 (49.2)	898 (48.3)	195 (53.9)	0.06
Maternal weight gain during pregnancy (kg)	9.4 ± 4.0	9.5 ± 4.1	8.8 ± 4.6	<0.05
Smoking status during pregnancy				
Never	1,221 (55.0)	1,008 (54.2)	213 (58.8)	0.23
Previously did, but quit before recognizing current pregnancy	461 (20.7)	396 (21.3)	65 (18.0)	
Previously did, but quit after finding out current pregnancy	326 (14.7)	275 (14.8)	51 (14.1)	
Yes, I still smoke	214 (9.6)	181 (9.7)	33 (9.1)	
Breastfeeding status at 2 years				
Currently continued breastfeeding	384 (17.3)	322 (17.3)	62 (17.1)	0.08
Stopped breastfeeding before	1,539 (69.3)	1,299 (69.8)	240 (66.3)	
Never breastfeeding	299 (13.5)	239 (12.8)	60 (16.6)	
Frequency of meal or snack per day at 2 years (≥3 times per day)	2,182 (98.2)	1834 (98.6)	348 (96.1)	<0.05
Height of mother (cm)	156.5 ± 5.4	157.0 ± 5.5	154.0 ± 5.8	<0.01
Height of father (cm)	170.1 ± 6.2	170.6 ± 6.7	167.4 ± 6.9	<0.01

^†^Small for gestational age defined by length and/or weight at birth <-2 standard deviation.

Data are reported in mean ± standard deviation or number (%).

p-values are from a t-test for continuous variables and a chi-squared test for categorical variables.

### Association Between Gestational Age-Adjusted Maternal TC Levels and No-CU Among SGA Infants

A total of 362 (16.3%) children with No-CU at 3 years were identified. After adjusting for Z-score of birth weight, age of mother at delivery, smoking status, weight gain during pregnancy, and study area as well as breastfeeding status and meal frequency at 2 years, OR and 95% CI for No-CU was 2.88 (1.32–6.31) in TC ≥95th percentile (≥294 mg/dl), compared to the reference group. Further adjustment for parents’ heights did not alter the association (OR: 2.95, 95% CI: 1.28–6.80) (**Table 2**). Sensitivity analysis using the complete data yielded similar results (**Tables 3**, **4**).

**Table 2:** Odds ratios of no-catch up growth at 3 years among infants born small for gestational age ^†^ at term according to percentile ranges of maternal serum total cholesterol value at mid-pregnancy^††^ (n = 2,222).

	Percentile range and values of maternal serum total cholesterol^††^ (mg/dL)									*p* for trend^†††^
	<5th	5th–9th	10th–14th	15th–19th	20th–79th	80th–84th	85th–89th	90th–94th	≥95th	
	(<180)	(180–189)	(190–197)	(198–203)	(204–260)	(261–267)	(268–277)	(278–293)	(≥294)	
All participants	(n = 171)	(n = 144)	(n = 146)	(n = 140)	(n = 1,251)	(n = 96)	(n = 93)	(n = 121)	(n = 60)	
Number of no-catch up growth at 3 years (%)	25 (14.6)	27 (18.8)	17 (11.6)	23 (16.4)	191 (15.3)	19 (19.8)	19 (20.4)	23 (19.0)	19 (31.7)	
Crude OR of SGA (95% CI)	0.95 (0.51–1.76)	1.25 (0.72–2.16)	0.72 (0.36–1.47)	1.06 (0.54–2.08)	Reference	1.32 (0.68–2.56)	1.40 (0.77–2.53)	1.29 (0.68–2.46)	2.54 (1.22–5.33)	0.08
Model 1	0.91 (0.49–1.70)	1.20 (0.69–2.08)	0.71 (0.35–1.44)	1.02 (0.51–2.02)		1.34 (0.69–2.60)	1.42 (0.78–2.57)	1.33 (0.69–2.54)	2.69 (1.27–5.73)	<0.05
Model 2	0.91 (0.48–1.73)	1.19 (0.68–2.07)	0.71 (0.34–1.45)	1.01 (0.51–2.03)		1.34 (0.69–2.60)	1.44 (0.78–2.65)	1.35 (0.70–2.63)	2.86 (1.32–6.23)	<0.05
Model 3	0.92 (0.49–1.74)	1.18 (0.68–2.06)	0.69 (0.33–1.45)	0.99 (0.49–2.00)		1.33 (0.68–2.06)	1.48 (0.80–2.75)	1.37 (0.70–2.65)	2.88 (1.32–6.31)	<0.05
Model 4	0.80 (0.41–1.57)	1.26 (0.70–2.26)	0.68 (0.32–1.47)	1.02 (0.48–2.14)		1.08 (0.53–2.20)	1.26 (0.65–2.42)	1.11 (0.54–2.27)	2.95 (1.28–6.80)	0.08

^†^Small for gestational age defined by length and/or weight at birth <-2 standard deviation.

^††^Maternal serum total cholesterol values were adjusted for gestational age (day) at blood sampling by residual methods.

^†††^p for trend estimated for the maternal serum total cholesterol percentile range as the following continuous variables: <5th (-4), 5th–9th (-3), 10th–14th (-2), 15th–19th (-1), 20th–79th (0), 80th–84th (1), 85th–89th (2), 90th–94th (3), ≥95th (4).

Model 1: adjustment for Z-score of birth weight and maternal age at delivery (year).

Model 2: adjustment for variables in model 1 + maternal weight gain during pregnancy (kg), smoking status during pregnancy (Never; Previously did, but quit before recognizing current pregnancy; Previously did, but quit after finding out current pregnancy; and Yes, I still smoke), and study areas (Hokkaido, Miyagi, Fukushima, Chiba, Kanagawa, Koshin, Toyama, Aichi, Kyoto, Osaka, Hyogo, Tottori, Kochi, Fukuoka, and South Kyushu/Okinawa).

Model 3: adjustment for variables in model 2 + breastfeeding status at 2 years (currently continued breast feeding, stopped breast feeding before, never breastfeeding) and frequency of meal or snack per day at 2 years (<3 times, ≥3 times).

Model 4: adjustment for variables in model 3 + maternal and paternal height (cm).

**Table 3:** Characteristics of participants by occurrence of catch-up growth at 3 years among infants born small for gestational age at term in the complete dataset (n = 1,134).

	Total	Catch-up growth at 3 years	No catch-up growth at 3 years	*p*-value
	(n = 1,134)	(n = 945)	(n = 189)	
Sex of child (male)	477 (42.1)	394 (41.7)	83 (43.9)	0.62
Gestational age at birth (weeks)	39.2 ± 1.1	39.3 ± 1.1	38.9 ± 1.1	<0.01
Z-score of length at birth	-2.0 ± 0.9	-1.9 ± 1.0	-2.1 ± 0.7	<0.05
Z-score of birth weight	-1.8 ± 0.8	-1.8 ± 0.8	-2.0 ± 0.8	<0.01
Age of mother at delivery (years)	31.4 ± 4.9	31.5 ± 4.9	31.1 ± 4.8	0.42
Parity (≥1)	512 (45.1)	420 (44.4)	92 (48.7)	0.30
Maternal weight gain during pregnancy (kg)	9.2 ± 3.9	9.3 ± 3.8	8.8 ± 4.1	0.13
Smoking status during pregnancy				
Never	693 (61.1)	569 (60.2)	124 (65.6)	0.38
Previously did, but quit before recognizing current pregnancy	237 (20.9)	206 (21.8)	31 (16.4)	
Previously did, but quit after finding out current pregnancy	138 (12.2)	114 (12.1)	24 (12.7)	
Yes, I still smoke	66 (5.8)	56 (5.9)	10 (5.3)	
Status on breastfeeding at 2 years			
Currently continued breastfeeding	201 (17.7)	167 (17.7)	34 (18.0)	0.75
Stopped before breastfeeding	789 (69.6)	661 (69.9)	128 (67.7)	
Never breastfeeding	144 (12.7)	117 (12.4)	27 (14.3)	
Frequency of meal or snack per day at 2 years (≥3 times per day)	1,112 (98.1)	932 (98.6)	180 (95.2)	< 0.05
Height of mother (cm)	156.7 ± 5.3	157.2 ± 5.2	154.2 ± 5.1	< 0.01
Height of father (cm)	170.0 ± 5.7	170.5 ± 5.6	167.3 ± 5.3	< 0.01

^†^Small for gestational age defined by length and/or weight at birth <-2 standard deviation.

Data are reported in mean ± standard deviation or number (%).

p-values are from a t-test for continuous variables and a chi-squared test for categorical variables.

**Table 4:** Odds ratios of no-catch up growth at 3 years among infants born small-for-gestational-age ^†^ at term according to percentile ranges of the maternal serum total cholesterol value at mid-pregnancy^††^ in the complete dataset (n = 1,134).

	Percentile range and values of maternal serum total cholesterol^††^ (mg/dL)									*p* for trend^†††^	
	<5th	5th–9th	10th–14th	15th–19th	20th–79th	80th–84th	85th–89th	90th–94th	≥95th	
	(<180)	(180–189)	(190–197)	(198–203)	(204–260)	(261–267)	(268–277)	(278–293)	(≥294)	
All participants	(n = 74)	(n = 76)	(n = 66)	(n = 65)	(n = 669)	(n = 54)	(n = 42)	(n = 61)	(n = 27)	
Number of no-catch up growth at 3 years (%)	13 (17.6)	16 (21.1)	8 (12.1)	11 (16.9)	101 (15.1)	10 (18.5)	8 (19.0)	12 (19.7)	10 (37.0)	
Crude OR of SGA (95% CI)	1.20 (0.64–2.26)	1.50 (0.83–2.71)	0.78 (0.36–1.67)	1.15 (0.58–2.27)	Reference	1.28 (0.62–2.62)	1.32 (0.60–2.94)	1.38 (0.71–2.68)	3.31 (1.47–7.43)	0.27
Model 1	1.15 (0.61–2.18)	1.40 (0.77–2.54)	0.76 (0.35–1.65)	1.09 (0.55–2.16)		1.34 (0.65–2.77)	1.43 (0.64–3.20)	1.44 (0.74–2.82)	3.76 (1.65–8.56)	0.13
Model 2	1.17 (0.61–2.24)	1.44 (0.78–2.64)	0.71 (0.33–1.55)	1.10 (0.54–2.15)		1.31 (0.63–2.74)	1.44 (0.64–3.26)	1.45 (0.73–2.86)	4.16 (1.77–9.75)	0.12
Model 3	1.17 (0.61–2.24)	1.45 (0.79–2.68)	0.72 (0.33–1.57)	1.05 (0.52–2.12)		1.33 (0.63–2.77)	1.50 (0.66–3.40)	1.48 (0.75–2.92)	4.31 (1.84–10.14)	0.11
Model 4	0.99 (0.50–1.98)	1.44 (0.75–2.77)	0.71 (0.31–1.59)	1.23 (0.59–2.56)		1.10 (0.51–2.36)	1.27 (0.54–3.01)	1.16 (0.57–2.37)	4.30 (1.79–10.37)	0.18

^†^Small for gestational age defined by length and/or weight at birth <-2 standard deviation.

^††^Maternal serum total cholesterol values were adjusted for gestational age (day) at blood sampling by residual methods.

^†††^p for trend estimated for maternal serum total cholesterol percentile range as following continuous variable: <5th (-4), 5th–9th (-3), 10th–14th (-2), 15th–19th (-1), 20th–79th (0), 80th–84th (1), 85th–89th (2), 90th–94th (3), ≥95th (4).

Model 1: adjustment for Z-score of birth weight and maternal age at delivery (year).

Model 2: adjustment for variables in model 1 + maternal weight gain during pregnancy (kg), smoking status during pregnancy (Never; Previously did, but quit before recognizing current pregnancy; Previously did, but quit after finding out current pregnancy; and Yes, I still smoke), and study areas (Hokkaido, Miyagi, Fukushima, Chiba, Kanagawa, Koshin, Toyama, Aichi, Kyoto, Osaka, Hyogo, Tottori, Kochi, Fukuoka, and South Kyushu/Okinawa).

Model 3: adjustment for variables in model 2 + breastfeeding status at 2 years (currently continued breast feeding, stopped breastfeeding before, never breastfeeding) and frequency of meal or snack per day at 2 years (<3 times, ≥3 times).

Model 4: adjustment for variables in model 3 + maternal and paternal height (cm).

**Figure 2:**
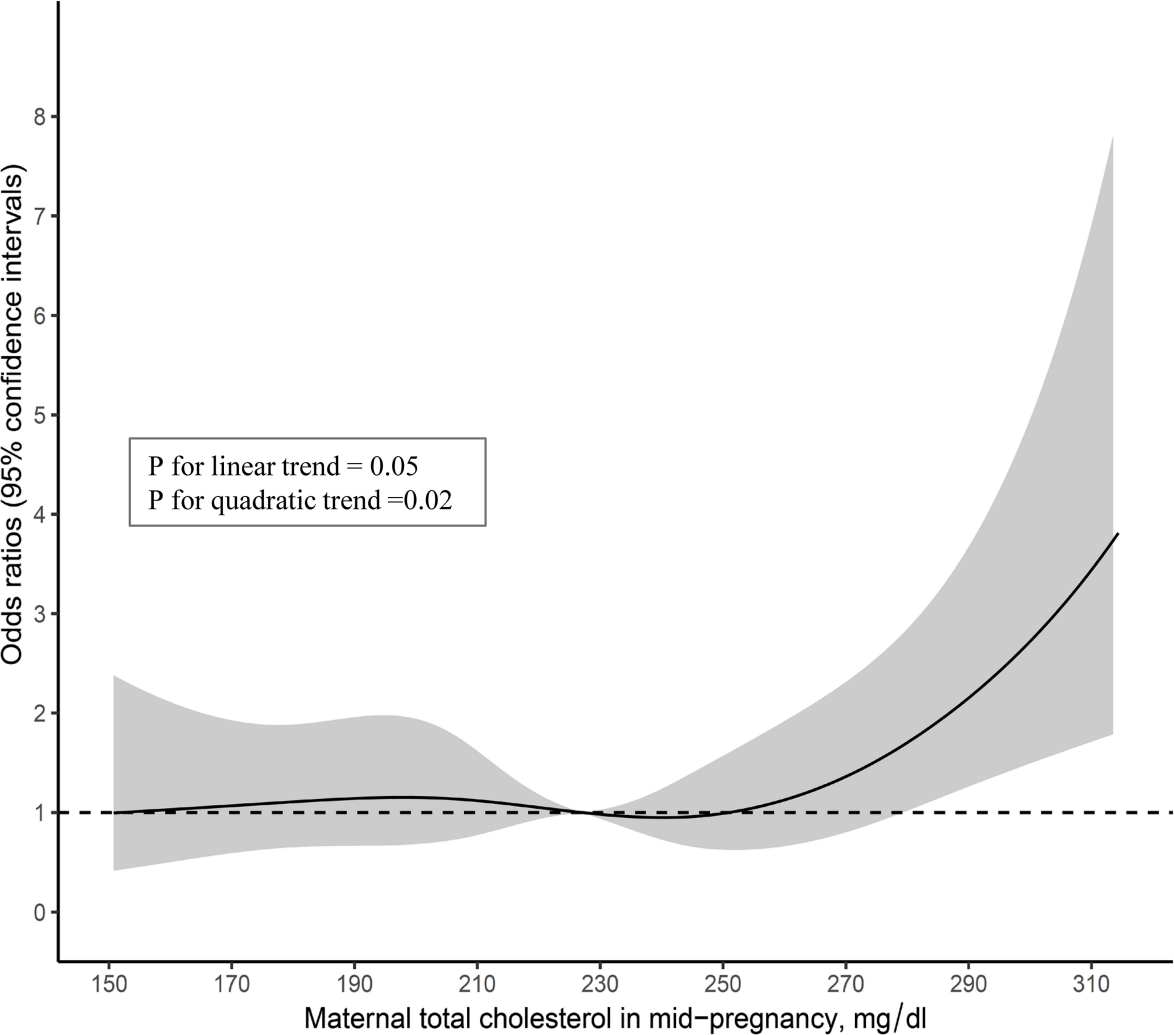
Multivariable-adjusted associations of maternal total cholesterol in mid-pregnancy with no-catch up growth in height at 3 years among infants born small-for-gestational-age at term (n=1,134).

The multiple-adjusted restricted cubic spline model using the complete data showed a non-linear trend of the association (p for linear trend = 0.05, p for quadratic trend = 0.02) (**Figure 2**).

Secondary analysis using the Japanese clinical definition for the diagnosis of SGA (n = 1,299) showed a total of 243 (18.7%) cases of No-CU at 3 years. The fully adjusted OR (95% CI) for No-CU was 3.71 (1.36–10.09) in the TC ≥95th percentile, compared to the reference group (table not shown).

## Discussion

The present study focused on 2,222 mother-and-SGA-infant pairs in a large-scale birth cohort study that revealed a significant association between high maternal TC and No-CU among SGA infants. To our knowledge, this is the first study to report a relationship between higher maternal TC at mid-pregnancy and No-CU at 3 years among SGA infants. Commencing GH treatment at the appropriate period in infancy or early childhood can help to improve catch-up growth and intellectual performances (15–17). Therefore, it is important to identify the predictors of No-CU in SGA, which can help judge the introduction of GH treatment. Previously, only umbilical cord miRNAs and bone maturation at 1 year have been reported as predictors of No-CU in SGA (48–50). In contrast, the predictive abilities of GH, the IGF-1 axis, and IGF binding protein-3 for No-CU have not been recognized (51). Our novel finding suggests that high maternal TC may inform clinical intervention with GH treatment and identify the SGA infants at high risk of future impairment of intellectual and psychological performance.

Our study showed that the cumulative incidence of No-CU at 3 years among full-term SGA infants was 16.3% using the global clinical definition of SGA for GH treatment: length and/or weight at birth <-2 SD (8). This is slightly higher than reports from Western countries, which indicated that 10%–15% of SGA infants had remained No-CU at 2 years of age (7, 8, 11). The difference might be explained by the varied reference values for the diagnosis of SGA or catch-up growth among previous studies (7). Using the Japanese clinical definition of SGA for GH treatment (i.e., length and/or weight at birth <-2 SD plus both length and weight at birth <10th percentile), a previous medical facility-based study implemented in Japan from 1998 to 2000 showed that 11% of 214 full-term infants were No-CU at 3 years of age (10). Another Japanese nationwide study since 2001 has indicated that 15.0% of 581 full-term SGA infants failed to catch up in height at 2.5 years of age (5). In contrast, our study results showed that 18.7% did not reach -2 SD in height before 3 years of age among 1,299 full-term SGA infants. However, the study population, period, data collection methods, and sample size differ among previous Japanese studies and ours; thus, the results may not be comparable. Considering the rising prevalence of low-birth-weight infants in Japan, as shown over the past three decades (52), the cumulative incidence of No-CU at a specific age among full-term SGA infants should be followed up as important epidemiological information.

A key finding of this study is that higher maternal TC (≥294 mg/dl) at mid-pregnancy is significantly associated with No-CU at 3 years of age among SGA infants and the multiple-adjusted restricted cubic spline model showed a non-linear trend. Based on these results, we speculate the presence of a threshold effect in the association of high maternal TC with No-CU among SGA infants. A further large-scale prospective study is required to confirm this association, given the small number of SGA children without CU, among participants in the highest maternal TC group, in the present study.

This is the first human study to report results that are consistent with those of previous animal experimental studies. A mini-review (31) reported that majority of rodent studies indicate that maternal high-fat diet adversely affects both embryonic bone development and bone volume in adult animals (30, 32, 33). In addition, Mikovic et al. indicated that high-fat maternal diet impaired offspring growth patterns and downregulated satellite cell activation and a healthy postnatal diet could not reverse any of these effects (34). The underlying mechanisms for the associations between high maternal TC levels and impairment of fetal and postnatal growth remain unclear. However, several researchers suspected that maternal high-fat diet induces low-grade inflammation (30) which leads to epigenetic modification in skeletal-myocyte proliferation (34). From another perspective, any hidden factors, such as exposure to environmental chemicals (53) or thyroid dysfunction (54, 55) may inhibit maternal lipid metabolism in peripheral tissues, leading to impairment of fetus and postnatal growth (56).

There are several limitations in the current study. First, the study lacked control for dietary intake after birth because of the absence of related data. Second, due to the missing values in our data, we used multiple-imputation analysis. However, according to the assumption of missing at random mechanism, the sensitivity analysis by the completer dataset showed no substantial difference. Third, the outcome data, height of children at 3 years of age, was obtained from a self-administrated questionnaire completed by the mother, and this might have created a response bias.

In conclusion, this study indicated that high maternal TC at mid-pregnancy was associated with No-CU among SGA infants. High maternal TC at mid-pregnancy among SGA infants could be an early alert for considering the need for GH treatment introduction. Furthermore, commencing GH treatment at the appropriate time may contribute to the improvement of long-lasting intellectual and psychological disabilities. This is the first human study to support previous animal experimental studies which indicated that maternal high-fat diet exposure induces impairment of growth and skeletal muscle development in the offspring. This finding could be a clue to elucidate the mechanism of no catch-up growth among SGA infants that leads to future intellectual and developmental disabilities. Future studies should elucidate the detailed mechanism underlying the effect of maternal high fat diet on growth and development of SGA infants.

## Data Availability Statement

The datasets presented in this article are not readily available because data are unsuitable for public deposition due to ethical restrictions and legal framework in Japan. It is prohibited by the Act on the Protection of Personal Information (Act No. 57 of 30 May 2003, amendment on 9 September 2015) to publicly deposit the data containing personal information. Ethical Guidelines for Medical and Health Research Involving Human Subjects enforced by the Japan Ministry of Education, Culture, Sports, Science and Technology and the Ministry of Health, Labour and Welfare also restricts the open sharing of the epidemiologic data. All inquiries about access to data should be sent to: jecs-en@nies.go.jp. The person responsible for handling enquiries sent to this e-mail address is Dr Shoji F. Nakayama, JECS Programme Office, National Institute for Environmental Studies. Requests to access the datasets should be directed to Dr Shoji F. Nakayama, jecs-en@nies.go.jp.

## Ethics Statement

The studies involving human participants were reviewed and approved by the Ministry of the Environment’s Institutional Review Board on Epidemiological Studies and the Ethics Committees in Japan. Written informed consent to participate in this study was provided by the participants’ legal guardian/next of kin.

## Members of the JECS Group as of 2022

Michihiro Kamijima (principal investigator, Nagoya City University, Nagoya, Japan), Shin Yamazaki (National Institute for Environmental Studies, Tsukuba, Japan), Yukihiro Ohya (National Center for Child Health and Development, Tokyo, Japan), Reiko Kishi (Hokkaido University, Sapporo, Japan), Nobuo Yaegashi (Tohoku University, Sendai, Japan), Koichi Hashimoto (Fukushima Medical University, Fukushima, Japan), Chisato Mori (Chiba University, Chiba, Japan), Shuichi Ito (Yokohama City University, Yokohama, Japan), Zentaro Yamagata (University of Yamanashi, Chuo, Japan), Hidekuni Inadera (University of Toyama, Toyama, Japan), Takeo Nakayama (Kyoto University, Kyoto, Japan), Tomotaka Sobue (Osaka University, Suita, Japan), Masayuki Shima (Hyogo Medical University, Nishinomiya, Japan), Hiroshige Nakamura (Tottori University, Yonago, Japan), Narufumi Suganuma (Kochi University, Nankoku, Japan), Koichi Kusuhara (University of Occupational and Environmental Health, Kitakyushu, Japan), and Takahiko Katoh (Kumamoto University, Kumamoto, Japan).

## Authors Contributions

KK conceptualized the design of the work, analyzed and interpreted the data, and drafted the manuscript. YI and HY contributed to the interpretation of data and revised the manuscript. TE, SK, TM, HT, HS, SS, MS-O, and JECS group members contributed to the acquisition of data and critically revised the manuscript for important content. MK led project administration and contributed to interpretation of data, and revising the manuscript. All authors provided final approval of the version to be published and agreed to be accountable for all aspects of the work in ensuring that questions related to the accuracy or integrity of any part of the work are appropriately investigated and resolved. All authors are in agreement with the content of the manuscript.

## Funding

This study was funded by the Ministry of the Environment, Japan. The findings and conclusions of this article are solely the responsibility of the authors and do not represent the official views of the above government.

## Conflict of Interest

The authors declare that the research was conducted in the absence of any commercial or financial relationships that could be construed as a potential conflict of interest.

## Publisher’s Note

All claims expressed in this article are solely those of the authors and do not necessarily represent those of their affiliated organizations, or those of the publisher, the editors and the reviewers. Any product that may be evaluated in this article, or claim that may be made by its manufacturer, is not guaranteed or endorsed by the publisher.

## Abbreviations

CI, confidence interval; GH, growth hormone; JECS, Japan Environment and Children’s Study; No-CU, no catch-up growth; OR, odds ratio; SD, standard deviation; SGA, small-for-gestational-age; TC, total cholesterol.
